# Effectiveness of a text-messaging-based smoking cessation intervention (“Happy Quit”) for smoking cessation in China: A randomized controlled trial

**DOI:** 10.1371/journal.pmed.1002713

**Published:** 2018-12-18

**Authors:** Yanhui Liao, Qiuxia Wu, Brian C. Kelly, Fengyu Zhang, Yi-Yuan Tang, Qianjin Wang, Honghong Ren, Yuzhu Hao, Mei Yang, Joanna Cohen, Jinsong Tang

**Affiliations:** 1 Department of Psychiatry, The Second Xiangya Hospital, Central South University, Changsha, China; 2 Mental Health Institute, The Second Xiangya Hospital, Central South University, Changsha, China; 3 National Clinical Research Center on Mental Disorders, Changsha, China; 4 National Technology Institute on Mental Disorders, Changsha, China; 5 Hunan Key Laboratory of Psychiatry and Mental Health, Changsha, China; 6 Department of Sociology, Purdue University, West Lafayette, Indiana, United States of America; 7 Center for Research on Young People’s Health, Purdue University, West Lafayette, Indiana, United States of America; 8 Global Clinical and Translational Research Institute, Bethesda, Maryland, United States of America; 9 Department of Psychological Sciences, Texas Tech University, Lubbock, Texas, United States of America; 10 Center for Advanced Study in the Behavioral Sciences, Stanford University, Stanford, California, United States of America; 11 Department of Drug Dependence, Shenzhen Mental Health Center, Shenzhen Kangning Hospital, Shenzhen, China; 12 Institute for Global Tobacco Control, Johns Hopkins Bloomberg School of Public Health, Baltimore, Maryland, United States of America; University of New South Wales, AUSTRALIA

## Abstract

**Background:**

China has the highest global prevalence of cigarette smokers, accounting for more than 40% of the total cigarette consumption in the world. Considering the shortage of smoking cessation services in China, and the acceptability, feasibility, and efficacy of mobile-phone-based text messaging interventions for quitting smoking in other countries, we conducted a mobile-phone-based smoking cessation study in China.

**Methods and findings:**

We conducted a randomized controlled trial in China across 30 cities and provinces from August 17, 2016, to May 27, 2017. Adult smokers aged 18 years and older with the intention to quit smoking were recruited and randomized to a 12-week high-frequency messaging (HFM) or low-frequency messaging (LFM) intervention (“Happy Quit”) or to a control group in a 5:2:3 ratio. The control group received only text messages unrelated to quitting. The primary outcome was biochemically verified continuous smoking abstinence at 24 weeks. Secondary outcomes included (1) self-reported 7-day point prevalence of abstinence (i.e., not even a puff of smoke, for the last 7 days) at 1, 4, 8, 12, 16, 20, and 24 weeks; (2) self-reported continuous abstinence at 4, 12, and 24 weeks; and (3) self-reported average number of cigarettes smoked per day. A total of 1,369 participants received 12 weeks of intervention or control text messages with continued follow-up for 12 weeks. The baseline characteristics of participants among the HFM (*n* = 674), LFM (*n* = 284), and control (*n* = 411) groups were similar. The study sample included 1,295 (94.6%) men; participants had a mean age of 38.1 (SD 9.79) years and smoked an average of 20.1 (SD 9.19) cigarettes per day. We included the participants in an intention-to-treat analysis. Biochemically verified continuous smoking abstinence at 24 weeks occurred in 44/674 participants in the HFM group (6.5%), 17/284 participants in the LFM group (6.0%), and 8/411 participants (1.9%) in the control group; participants in both the HFM (odds ratio [OR] = 3.51, 95% CI 1.64–7.55, *p <* 0.001) and the LFM (OR = 3.21, 95% CI 1.36–7.54], *p* = 0.002) intervention groups were more likely to quit smoking than those in the control group. However, there was no difference in quit rate between the HFM and LFM interventions. We also found that the 7-day point quit rate from week 1 to week 24 ranged from approximately 10% to more than 26% with the intervention and from less than 4% to nearly 12% without the intervention. Those who continued as smokers in the HFM group smoked 1 to 3 fewer cigarettes per day than those in the LFM group over the 24 weeks of trial. Among study limitations, the participants were able to use other smoking cessation services (although very few participants reported using them), cotinine tests can only detect smoking status for a few days, and the proportion of quitters was small.

**Conclusions:**

Our findings demonstrate that a mobile-phone-based text messaging intervention (Happy Quit), with either high- or low-frequency messaging, led to smoking cessation in the present study, albeit in a low proportion of smokers, and can therefore be considered for use in large-scale intervention efforts in China. Mobile-phone-based interventions could be paired with other smoking cessation services for treatment-seeking smokers in China.

**Trial registration:**

ClinicalTrials.gov NCT02693626.

## Abstract 2

Jinsong Tang and colleagues report on a randomized trial done in China, aiming to evaluate a smoking cessation intervention based on texting.

## Author summary

**Why was this study done?:**

- China has the highest prevalence of cigarette smokers, accounting for more than 40% of the total cigarette consumption in the world.
- Smoking cessation remains the single most effective prevention measure for lung cancer and other smoking-related disorders and health conditions.
- The availability of smoking cessation services in China is extremely limited.
- Mobile-phone-based text messaging interventions for quitting smoking have proven cost-effective in other countries.

**What did the researchers do and find?:**

- We conducted a randomized controlled trial across various cities and provinces in China.
- The “Happy Quit” intervention consisted of text messages based on the principles of cognitive behavioral therapy. Messages were aimed at improving self-efficacy and behavioral capability for quitting.
- A total of 1,369 participants—674 in the high-frequency messaging group, 284 in the low-frequency messaging group, and 411 in the control group—received 12 weeks of intervention or control text messages, with an additional 12 weeks of follow-up.
- We found that biochemically verified continuous smoking abstinence at 24 weeks was 6.5% in the high-frequency messaging group, 6.0% in the low-frequency messaging group, and 1.9% in the control group.

**What do these findings mean?:**

- The findings of our trial suggest that a mobile-phone-based text messaging intervention (Happy Quit), with either high- or low-frequency messaging, is effective for Chinese cigarette smokers.
- This intervention should be considered for inclusion with smoking cessation services, and could be made widely available for smokers seeking treatment in China.

## Introduction

Cigarette smoking, as a major public health problem, remains the leading preventable cause of death and disability in China and other countries. In 2015, it was estimated that there were 933.1 million daily smokers worldwide, and more than 6 million deaths (accounting for 11.5% of global deaths) were attributable to cigarette smoking, including at least 1 million in China [1]. Over 80% of deaths attributable to smoking were among men, and more than half of all cigarette smoking took place in 4 countries (China, India, the US, and Russia) [2], with China having the highest proportion of smokers, accounting for more than 40% of the world’s total cigarette consumption [3]. In comparison with many countries, especially European countries, smoking remains highly normalized within China, further necessitating the advancement of smoking cessation interventions for Chinese smokers.

Nationwide prospective studies show that “the annual number of deaths in China that are caused by tobacco will rise from about 1 million in 2010 to 2 million in 2030 and 3 million in 2050, unless there is widespread cessation” [1]. While smoking cessation remains the single most effective prevention measure for lung cancer and other smoking-related diseases [4], the availability of smoking cessation services is extremely limited in China, and the majority of cessation attempts are not successful [5]. Insufficient smoking cessation services are an important contributing factor for the low cessation rates reported in China. A study conducted in 21 Chinese cities reported that almost half of smokers would like to stop [6]. However, the rate of smoking cessation among smokers in urban areas, which have the highest rate of successful quitting, was just 10% to 13% according to previous studies in 2010 and 2014 [6,7]. There is an urgent need to improve the availability and utilization of smoking cessation interventions and to reach underserved populations in China.

Evidence indicates that individualized counseling can effectively assist smokers to stop smoking [8]. However, less than one-third of physicians in China believe that most smokers will follow their cessation advice [9], and, in fact, many smokers do not receive smoking cessation advice or medications from their physicians [5]. Furthermore, a study showed that less than half of physicians usually ask about patients’ smoking status, and less than 7% of physicians set quit dates or prescribe pharmaceutical therapies to help smokers quit [9]. Self-help smoking cessation materials have the potential to reach large numbers of smokers, but the effect is likely to be small [10]. An important unmet need in China is the dissemination of an effective smoking cessation intervention that can reach a large population regardless of social class, income, educational status, or geographic region. Mobile-phone-based interventions may hold such promise.

Mobile phones are widely used in China, and most of these phones are smartphones. By the end of 2017, the number of mobile phone subscribers had reached 1.4 billion in China (https://www.statista.com/statistics/278204/china-mobile-users-by-month/). Mobile-phone-based interventions such as those employing short message service (SMS) communications have been used to intervene regarding risky behaviors, improve preventive healthcare, and promote self-management of chronic illness. For preventive healthcare, strong evidence exists that interventions may be successfully delivered through SMS [11]. Mobile-phone-based text messaging for intervention in smoking cessation has several potential benefits, such as easy access, cost-effective delivery, independence from time and place, and the potential to help a diverse range of smokers quit, particularly those who may not otherwise have easy access to cessation services.

In view of a shortage of smoking cessation services in China and evidence of the feasibility, acceptability, and efficacy of mobile-phone-based text messaging interventions for quitting smoking from other countries [12,13], we designed a population-based, widely accessible smoking cessation program in China. In this single-blind randomized trial of a mobile-phone-based text messaging intervention (“Happy Quit”), the primary objective was to test the effectiveness of the Happy Quit program in the general population within China. In particular, we were interested in testing the efficacy of Happy Quit on biochemically verified continuous smoking abstinence at 24 weeks (primary outcome). Based on the efficacy of similar interventions in previous studies in other countries, we hypothesized that Happy Quit can be applied as a promising and innovative means to deliver smoking cessation services in China. Mobile-phone-based text messaging interventions such as Happy Quit could represent an important, effective, feasible, and affordable—but as yet little used—medium to provide nationwide smoking cessation support services.

### Evidence before this study

We searched databases (China National Knowledge Infrastructure, Wanfang Data resources, Google Scholar, Embase, Cochrane, Medline, and PsycInfo) for studies of mobile-phone-based text messaging interventions for smoking cessation conducted from 1980 to February 2018. Randomized controlled trials (RCTs) that compared a behavioral smoking cessation intervention delivered via mobile phone with a no-intervention comparison group among general cigarette smokers were included. We searched for mobile-phone-based text messaging interventions for smoking cessation without language restrictions. A literature review indicated that there were few RCTs of smoking cessation text messaging interventions with biochemically verified continuous smoking abstinence at 6 months as an outcome. A large study from the UK showed that biochemically verified continuous abstinence at 6 months was 10.7% in the text messaging intervention group and 4.9% in the control group (relative risk = 2.20, 95% CI 1.80–2.68, *p <* 0.001) [14]. Another RCT conducted in the US reported that biochemically confirmed repeated point prevalence of abstinence also favored the intervention group, with 11.1% abstinent compared to 5.0% of the control group (relative risk = 2.22, 95% CI 1.16–4.26, *p <* 0.05) [15]. Other trials reported higher abstinence with text messaging in other contexts. For example, one trial from Spain that assessed text messaging as an adjunct to health advice in smoking cessation reported that 24.4% of patients in the intervention group and 11.9% of the control participants had stopped smoking at 6 months (relative risk = 2.05, 95% CI 1.24–3.39, *p* = 0.004) [16]. However, China is distinct as a country that consumes more cigarettes than all other low- and middle-income countries combined (http://www.tobaccoatlas.org/topic/cigarette-use-globally/) and has very limited smoking cessation services. Thus, the aim of the study was to assess the effectiveness of a phone-based text messaging intervention (Happy Quit) for smoking cessation in China.

## Methods

### Theoretical basis of the Happy Quit intervention

The development of the Happy Quit program was based on cognitive behavioral therapy (CBT) theory, which hypothesizes that people’s emotions, behaviors, and physiology are influenced by their perception of events [17]. Messages were aimed at improving self-efficacy for quitting, describing outcome expectations from quitting, increasing perceived social support for quitting, modeling effective quitting strategies and coping skills, and increasing behavioral capability for quitting.

We implemented the Happy Quit intervention by combining the SMS format with CBT approaches. The detailed implementation plan was summarized and described in a previous publication [18]. A crucial component of developing a smoking cessation plan is setting a quit date and making a strong personal commitment to quit on that day, which increases the chances of quitting for good. Text-messaging interventions can reach large groups of people at a very low cost per person. Furthermore, text messages have the potential to incorporate qualities often linked with effective health communication interventions, such as interactivity, personalization, tailoring, and message repetition [19].

### Study design and participants

We conducted a single-blind randomized trial of Happy Quit—individualized smoking cessation services that support cessation via mobile phone text messages. The allocation was unknown to all investigators. Intervention staff who delivered the intervention did not take outcome measurements. All investigators were kept masked to outcome data. This trial was conducted in China, and 1,369 participants were randomized between August 17, 2016, and May 27, 2017. The published protocol describes procedures in detail [18]. In brief, participants were daily smokers 18 years of age and older living in China. Additional eligibility criteria included the following: being able to read and write in Chinese, owning a text-capable cell phone and knowing how to text, being willing to make an attempt to quit smoking in the next month, agreeing to smoking cessation status verification by a significant other (e.g., family member, friend), and being willing to provide informed consent to participate in the study. Given that this study was mainly performed with text messaging, no restrictions on setting or location were needed.

This trial was approved by the Second Xiangya Hospital of Central South University Review Board (No. S007 [2015] and No. S111 for adding the low-frequency messaging intervention [2016]), and the study was carried out in accordance with the Declaration of Helsinki. The Consolidated Standards of Reporting Trials (CONSORT) checklist (S1 CONSORT Checklist) and protocol (S1 Study Protocol) are provided. Preceding the study, we conducted the initial screening and the orientation session through telephone call; participants were required to know about the consent form and could ask any questions they had about it. After the study had been fully explained and questions had been answered, and before the first intervention or control text message was sent, the participants were sent an agreement of consent form by text message. The consent form outlined the procedures to be followed. Participants were fully informed about the purpose, procedures, and measurements of the study. For additional ethics approval and consent details, see the study protocol [18].

A change of adding a low-frequency text messaging intervention group was made while the trial was underway. As some participants in the pilot stage requested less frequent messages—and a text messaging smoking cessation study for Chinese Nokia Life Tools subscribers showed efficacy for both high- and low-frequency text messaging interventions [20]—we recruited a group of participants for a low-frequency text messaging intervention not described in the protocol. This permitted an assessment of whether the intervention might be efficacious with reduced frequency of delivery.

### Randomization and masking

In this single-blind RCT, participants were randomly allocated to 1 of 3 conditions in a 5:2:3 ratio: a high-frequency messaging (HFM) intervention, a low-frequency messaging (LFM) intervention, or the control group. In the previously published protocol, we specified that participants would be randomly allocated to the messaging intervention or control groups in a 1:1 ratio. After discussing the frequency of messages with some participants and specialists during the initial stage of the study, we made a change in the trial by adding the LFM group to test the efficacy of the intervention for quitting smoking at a reduced frequency of delivery; we split the original control group into a LFM group and a control group by a 2:3 ratio, which led to the overall 5:2:3 allocation ratio.

Participants were randomly allocated using an independent telephone randomization system that included a minimization algorithm balancing for sex (male, female), age (18–34 years, >34 years), educational level (years of education: ≤12 years, >12 years), and Fagerstrom Test for Nicotine Dependence (FTND) score (≤5, >5). The Happy Quit message delivery system automatically generated HFM or LFM intervention or control texts according to the allocation. Participants, investigators, and research personnel were masked to treatment allocation. Control participants are likely to have suspected their allocation as they only received text messages unrelated to quitting.

### Procedures

#### Recruitment

We advertised this service to smokers on the radio, bus billboards, online (e.g., websites, QQ, WeChat) as well as in newspapers, hospitals, and pharmacies. Potential participants registered their interest by sending a text message, which also indicated that they had regular access to a mobile phone. Research assistants then called each respondent by telephone to assess eligibility and collect baseline data. Research assistants explained the study to each participant and told them that they would be allocated to either a control group or to a group that receives the Happy Quit program. Participants who enrolled in this study could withdraw from Happy Quit at any time by sending a “退出 [Pinyin: Tuì-chū; English: withdrawal or stop]” message. Participants were free to participate in any other smoking cessation service or support they wished to, although the availability of such services is low in China. Information on use of other cessation services was collected during screening. For those participants who did not meet eligibility criteria or declined to participate, research assistants provided our contact information (phone number and email address) to them.

#### Text messaging library

A text message library was developed with the input of smokers and smoking cessation professionals. The intervention texts included motivational messages and behavior change techniques. Messages encouraged participants to persevere with the quit attempt and focus on their success so far. The motivational messages provided positive feedback and emphasized the benefits achieved by quitting, as well as providing information about the consequences of smoking, how to quit and remain abstinent, and how others would approve of successful abstinence. The behavior changing messages prompted participants to get rid of cigarettes, ashtrays, and lighters and to avoid environments where they would normally smoke, and encouraged participants to identify the challenges of quitting and plan how to overcome them. This content also covered information relevant to quitting—e.g., symptoms to expect on quitting, tips to cope with craving, tips to avoid weight gain and improve nutrition, advice on avoiding smoking triggers, instructions on breathing exercises to perform instead of smoking, and motivational support and distraction. Examples of behavior change techniques or strategies included minimizing motivation to smoke before the quit date, maximizing motivation to remain abstinent following the quit date, and promoting activities to reduce exposure to smoking cues. Messages included the following: “Time for a Mini-Quit challenge. For the next 4 hours, stay away from cigs. Practice dealing with cravings without smoking.”; “Cravings last less than 5 minutes on average. To help distract yourself, try sipping a drink slowly until the craving is over.”; “Look for cues or triggers for your smoking. For each one, write down something you can do instead, e.g., if you’re angry, try deep breathing.”; and “Stay away from people/places that make you think of smoking. You will find it easier to cope that way and you will avoid secondhand smoke.” The messages were developed by a multidisciplinary team including current smokers, ex-smokers, health researchers, and experts in CBT and smoking cessation. The Happy Quit program also provided personalized messages. Participants in the intervention groups could request on-demand support, e.g., by texting “应对渴求 [Pinyin: Yìng-duì-kě-qiú; English: tips for crave]”, “调整心情 [Pinyin: Tiáo-zhěng xīn-qíng; English: tips for mood]”, or “为什么戒烟 [Pinyin: Wèi-shé-me-jiè-yān; English: why quit]” to get 24-hour, toll-free support.

#### Intervention

We initially recruited 200 participants to assess the Happy Quit program with a 4-week follow-up point after quitting. As some participants preferred less frequent messages, we added the LFM group. Then, we invited 34 participants, 17 in the HFM and 17 participants in the LFM group, to assess the program; both HFM and LFM messaging were acceptable (detailed information in S1 Table).

A quit date was negotiated with all participants by sending text messages; the quit date was then confirmed by research assistants with phone calls. Participants in both the intervention groups and control group were asked to set a quit date within 1 month of randomization and were encouraged to select a quit date about 2 weeks from the welcome day if they had no disagreement with it. For the HFM group, 3 to 5 messages were sent per day for the time leading up to the quit day and the following 12 weeks (12-week intervention period). On the quit day, a free month of outgoing text messaging was given to the participants in all groups, with participants encouraged to tell their close friends and family they were quitting on that day. This process encouraged communication and support, and also helped with distraction, given the time spent in writing and receiving text messages. For the LFM group, 3 to 5 messages were sent per week for the time leading up to the quit day and the following 12 weeks (12-week intervention period). After 12 weeks, the intervention became much less intensive, with the number of sent text messages reduced to 3 to 5 per week for the HFM group and 1 to 2 per week for the LFM group for the next 12 weeks (12-week follow-up period). These messages focused on maintaining cessation among those who had quit and encouraged those who had reduced smoking, and provided tips for craving, mood, weight management, and reasons to quit through 24 weeks. Control group participants only received 1 text message every week, thanking them for being in the study, providing study center contact details, and reminding them of the time until the end of follow-up. After completion of the trial, we offered the Happy Quit program booklet to each participant.

Demographic and smoking characteristics were assessed for each participant at baseline. All participants were assessed at 1, 4, 8, 12, 16, 20, and 24 weeks for continuous smoking abstinence, point prevalence of abstinence, and how many cigarettes smoked per day during the past week, if they were still smoking. In addition to text message responses, 7-day point prevalence of abstinence was also assessed by brief telephone interviews at each time point. A phone call was made to their significant other (e.g., family member, friend) to further confirm their quit status. Participants were rewarded with a 40 Chinese yuan (CNY) mobile-phone-based payment (whether they quit or not) each month, and a reminder of the benefit was sent to the participants at each assessment point. The participants who self-reported continuous abstinence at 24 weeks were invited to provide a urine sample for biochemical verification. After 24 weeks, cotinine (nicotine metabolite) urine dipsticks and 20 CNY in cash was mailed to each participant who self-reported 24 weeks of continuous abstinence, for determination of smoking status. Participants were requested to do the test twice—that day and the next day, with their significant other confirming that the participant took the test—before sending a photo of the resulting dipsticks to us by WeChat (the most popular Chinese multipurpose messaging, social media, and mobile payment app). They received the 20 CNY cash for biochemical verification irrespective of the result of the verification. At the end of the study, we thanked every participant and provided our contact information (including phone number, email address, and WeChat ID) to them.

### Outcome measures

The primary outcome was biochemically verified continuous smoking abstinence at 24 weeks. Continuous smoking abstinence at 24 weeks was defined as smoking not more than 5 cigarettes from the quit day to 24 weeks. Participants who smoked more than 5 cigarettes during this period were considered as having relapsed [21]. We applied a urine cotinine cutoff point of 200 ng/ml because it has been commonly used to validate self-reported smoking status, with more than 95% accuracy [22]. Secondary outcomes included (1) self-reported 7-day point prevalence of abstinence (not even a puff of smoke, for the last 7 days) at 1, 4, 8, 12, 16, 20, and 24 weeks; (2) self-reported continuous abstinence at 4, 12, and 24 weeks; and (3) self-reported average number of cigarettes smoked per day.

### Statistical analysis

#### Power estimation

On the basis of the results of the intervention review paper [13] and the study with the largest sample size published in *The Lancet* [14], we estimated that biochemically verified continuous smoking abstinence at 24 weeks would be around 5% in the control group and 10% in the intervention groups. With this estimate and a 5% 2-sided type I error rate, a power of 80% requires a sample size of 864 and a power of 90% requires a sample size of 1,158 (per the Sealed Envelope power calculator, 2012). Therefore, we reduced our recruitment target from 2,000, and ended with 1,369 participants. We calculated that the study sample size of 1,369 participants would have adequate power at 90% to detect a significant difference.

Statistical analyses were conducted with R software (https://www.r-project.org/) and SPSS version 22 (IBM). For determination of smoking abstinence rate, an intention-to-treat analysis was used. This type of analysis is considered the most conservative and is the standard for smoking cessation studies [13]. Thus, all participants were analyzed in the study arm to which they were randomized. As dropout rates were higher in the 2 intervention arms than in the control arm, and the participants with unknown smoking status were assumed to have continued smoking, this results in conservative estimates of intervention efficacy.

Seven-day abstinence and continuous abstinence were compared between participants in the intervention groups and control group at week 24 after the quit date using a mixed-effects model. The independent variables were each intervention versus the control condition, assessment points, and covariates identified in preliminary analyses. We also calculated quit rates (including biochemically verified continuous abstinence at 24 weeks; self-reported continuous abstinence at 4, 12, and 24 weeks; and self-reported 7-day point prevalence of abstinence at 1, 4, 8, 12, 16, 20, and 24 weeks). Odds ratios (ORs) were used to measure the outcomes for the intervention groups (both HFM and LFM) compared with the control group, and χ^2^ tests were used to test for statistical significance. We compared the number of cigarettes consumed per day during the intervention and follow-up periods between the HFM group and LFM group by 2-sample *t* test. We used Kaplan–Meier curves for analyses of time to relapse. All tests were 2-tailed. A 2-sided *p <* 0.05 was used to determine statistical significance.

## Results

**Fig 1:**
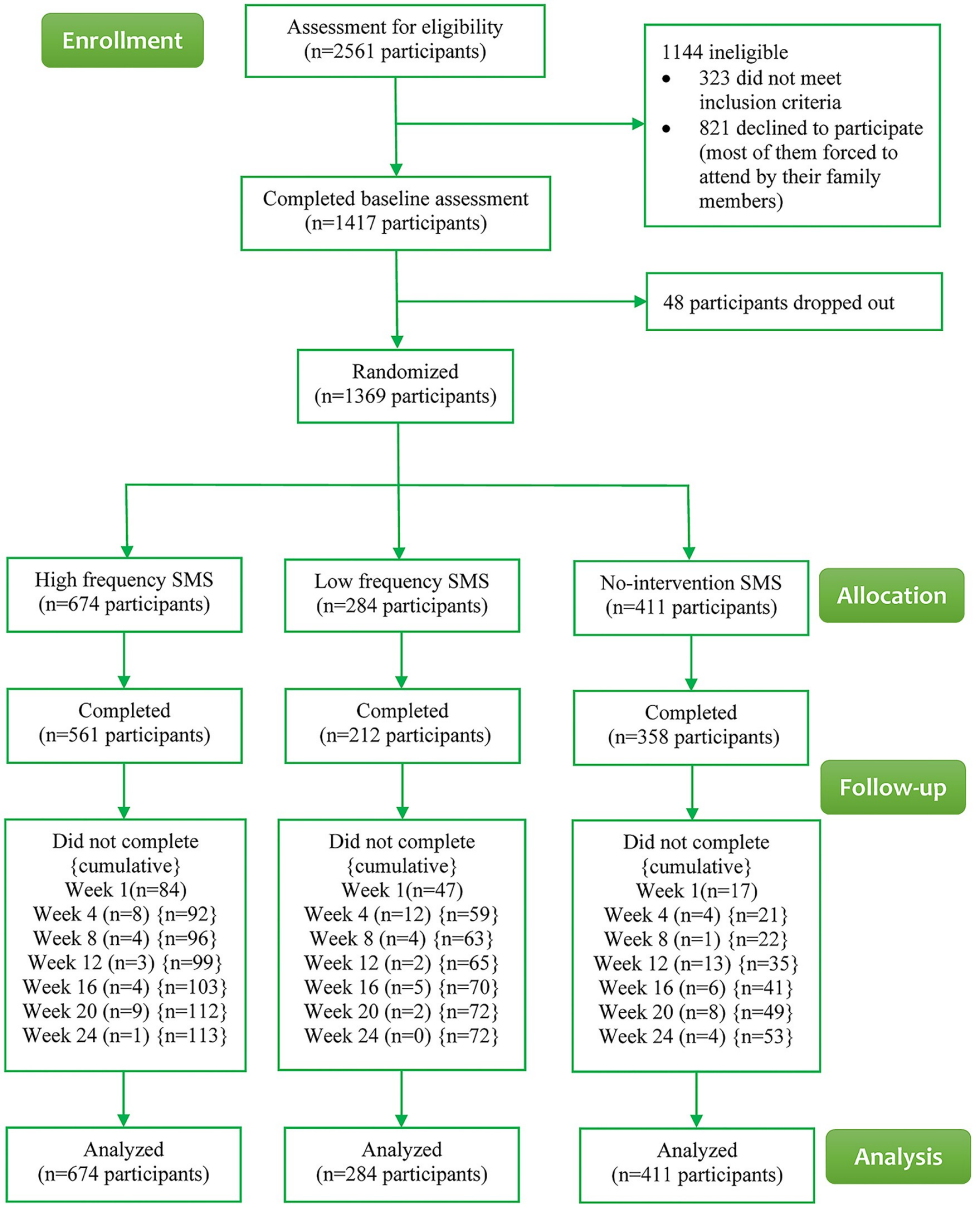
Flowchart of participants. SMS, short message service.

Fig 1 shows the flowchart of participants who were screened and participated in the study intervention and follow-up periods. There were 2,561 individuals who were assessed for eligibility between August 17, 2016, and May 27, 2017, of which 1,144 were ineligible, leaving 1,417 eligible for inclusion in this study. Forty-eight were eligible but declined to participate. A total of 1,369 participants were randomly assigned within the trial, with 674 in the HFM group, 284 in the LFM group, and 411 in the control group. At the end of the trial, 1 participant reported using other cessation services, and he was still smoking. By the end of the 24-week trial period, the trial was completed by 83.2%, 74.6%, and 87.1% of participants in the HFM group, LFM group, and control group, respectively. The rates of completion were similar in the HFM group and the control group (*p* = 0.087). However, the LFM group had a lower completion rate than either the HFM or control group (*p <* 0.001). The geographical distribution of participants in China is shown in S1 Table.

Table 1 shows the demographics and smoking characteristics for all participants. Treatment groups were evenly balanced for baseline characteristics. Overall, the study sample included 1,295 (94.6%) men, and participants had a mean age of 38.1 (SD 9.79) years. Participants smoked an average of 20.1 (SD 9.19) cigarettes per day, with an average FTND score of 4.6 (SD 2.16). The average number of previous attempts to quit was 1.5 (SD 4.59), and 786 (57.4%) participants had made at least 1 previous attempt to quit smoking.

**Table 1:** Baseline characteristics of study groups.

Characteristic	HFM group (%)	LFM group (%)	Control group (%)
**Total**	674	284	411
**Sex**			
Male	641 (95.1%)	267 (94.0%)	387 (94.2%)
Female	33 (4.9%)	17 (6.0%)	24 (5.8%)
**Age (years), mean (SD)**	38.1 (9.74)	37.2 (9.79)	38.7 (9.83)
**Age (years)**			
18–34	258 (38.3%)	124 (43.7%)	156 (38.0%)
35+	416 (61.7%)	160 (56.3%)	255 (62.0%)
**Education (years)**			
≤12	163 (24.2%)	77 (27.1%)	109 (26.5%)
>12	511 (75.8%)	207 (72.9%)	302 (73.5%)
**Number of cigarettes smoked per day, mean (SD)**	20.3 (9.49)	19.8 (8.84)	20.0 (8.93)
**Number of cigarettes smoked per day**			
≤10	114 (16.9%)	53 (18.7%)	77 (18.7%)
11–20	399 (59.2%)	162 (57.0%)	238 (57.9%)
21–30	103 (15.3%)	53 (18.7%)	64 (15.6%)
≥30	58 (8.6%)	16 (5.6%)	32 (7.8%)
**Number of previous quit attempts, mean (SD)**	1.5 (4.84)	1.4 (2.52)	1.6 (5.22)
**Number of previous quit attempts**			
Never	287 (42.6%)	126 (44.4%)	170 (41.4%)
1–5 times	370 (54.9%)	149 (52.5%)	225 (54.7%)
≥6 times	17 (2.5%)	9 (3.2%)	16 (3.9%)
**FTND score, mean (SD)**	4.7 (2.19)	4.5 (2.10)	4.6 (2.17)
**FTND score**			
<4 (minimally dependent)	202 (30.0%)	88 (31.0%)	128 (31.1%)
4–6 (moderately dependent)	328 (48.7%)	141 (49.6%)	203 (49.4%)
>6 (highly dependent)	144 (21.4%)	55 (19.4%)	80 (19.5%)

Data are *n* (%) unless otherwise indicated.

FTND, Fagerstrom Test for Nicotine Dependence; HFM, high-frequency messaging; LFM, low-frequency messaging.

Regarding the primary outcome, biochemically verified continuous smoking abstinence at 24 weeks was significantly higher in both the HFM (6.5% versus 1.9%, *p* < 0.001) and LFM (6.0% versus 1.9%, *p* = 0.002) groups compared with the control group (see Table 2 and S2 Fig). However, there was no significant difference in the rate of biochemically verified continuous smoking abstinence between the HFM and LFM groups (6.5% versus 6.0%, *p* = 0.754).

**Table 2:** Verified continuous smoking abstinence and 7-day point prevalence (intention-to-treat) by group.

Outcome	Control participants (%) (*n* = 411)	HFM participants			LFM participants		
		Participants (%) (*n* = 674)	OR (95% CI)	*p*-Value*	Participants (%) (*n* = 284)	OR (95% CI)	*p*-Value*
**Primary outcome**							
Verified abstinence	8 (1.9%)	44 (6.5%)	3.51 (1.64–7.55)	<0.001	17 (6.0%)	3.21 (1.36–7.54)	0.002
**Secondary outcomes**							
Self-reported continuous abstinence							
4 weeks	11 (2.7%)	63 (9.4%)	3.74 (1.95–7.20)	<0.001	21 (7.4%)	2.90 (1.38–6.12)	0.004
12 weeks	9 (2.2%)	56 (8.3%)	4.05 (1.98–8.27)	<0.001	18 (6.3%)	3.02 (1.34–6.83)	0.006
24 weeks	8 (1.9%)	46 (6.8%)	3.69 (1.72–7.90)	<0.001	18 (6.3%)	3.41 (1.46–7.95)	0.004
Self-reported 7-day point prevalence of abstinence							
1 week	15 (3.6%)	69 (10.2%)	3.01 (1.70–5.34)	<0.001	23 (8.1%)	2.33 (1.19–4.54)	0.004
4 weeks	24 (5.8%)	88 (13.1%)	2.42 (1.51–3.87)	<0.001	33 (11.6%)	2.12 (1.22–3.67)	<0.001
8 weeks	44 (10.7%)	113 (16.8%)	1.68 (1.16–2.44)	0.002	41 (14.4%)	1.41 (0.89–2.22)	0.020
12 weeks	28 (6.8%)	138 (20.5%)	3.52 (2.30–5.40)	<0.001	56 (19.7%)	3.36 (2.07–5.44)	<0.001
16 weeks	47 (11.4%)	177 (26.3%)	2.76 (1.95–3.91)	<0.001	59 (20.8%)	2.03 (1.34–3.08)	<0.001
20 weeks	48 (11.7%)	163 (24.2%)	2.41 (1.70–3.42)	<0.001	69 (24.3%)	2.42 (1.62–3.64)	<0.001
24 weeks	27 (6.6%)	130 (19.3%)	3.40 (2.20–5.25)	<0.001	55 (19.4%)	3.42 (2.10–5.57)	<0.001

ORs and *p*-values are for comparison with the control group.

*Bonferroni corrected *p*-values.

HFM, high-frequency messaging; LFM, low-frequency messaging; OR, odds ratio.

Regarding secondary outcomes, self-reported continuous abstinence at 4, 12, and 24 weeks and self-reported 7-day point prevalence of abstinence at 1, 4, 8, 12, 16, 20, and 24 weeks are displayed in Table 2 and S1 Fig. Both secondary outcomes at all time points were significantly better in the HFM and LFM groups than the control group, except for self-reported 7-day point prevalence of abstinence at 8 weeks in the LFM group.

Given that the demographic characteristics of the HFM and LFM groups were similar, we combined these together as a unified intervention group, and compared it with the control group. The baseline characteristics of the combined intervention group and control group are shown in S3 Table. The results for continuous smoking abstinence and 7-day point prevalence (intention-to-treat) are shown in S4 Table. Biochemically verified continuous smoking abstinence at 24 weeks was again significantly higher in the combined intervention group than in the control group (6.4% versus 1.9%, *p <* 0.001). The rate of self-reported continuous abstinence at 4, 12, and 24 weeks and self-reported 7-day point prevalence of abstinence at 1, 4, 8, 12, 16, 20, and 24 weeks were significantly higher in the combined intervention group than in the control group (*p <* 0.001 at each assessment point, except for self-reported 7-day point prevalence of abstinence at 8 weeks, with *p* = 0.011); for more details, see S4 Table.

For those who continued smoking, Table 3 lists the average number of cigarettes consumed per day from week 1 to week 24 for the HFM group and LFM group. The number of cigarettes smoked per day was not different across all assessment points, except that participants in the HFM group smoked fewer cigarettes per day than those in the LFM group at 8 weeks and 12 weeks.

**Table 3:** Cigarettes consumed* per day during intervention and follow-up period by intervention group.

Assessment point	HFM group		LFM group		*p*-Value
	*n*	Mean (SD)	*n*	Mean (SD)	
1 week	605	14.9 (8.78)	261	15.1 (8.81)	0.735
4 weeks	586	13.0 (8.17)	251	14.3 (8.77)	0.037
8 weeks	561	11.0 (8.58)	243	13.3 (8.68)	<0.001
12 weeks	536	10.2 (9.00)	228	13.4 (8.72)	<0.001
16 weeks	497	12.7 (8.08)	225	13.6 (8.96)	0.176
20 weeks	511	12.6 (7.88)	215	14.2 (8.65)	0.017
24 weeks	544	12.5 (8.21)	229	13.5 (8.40)	0.123

Only includes those who self-reported smoking at least 1 cigarette in past 7 days.

*Self-reported average number of smoked cigarettes per day in the past 7 days.

HFM, high-frequency messaging; LFM, low-frequency messaging.

## Discussion

The purpose of this trial was to develop and evaluate a widely applicable smoking cessation program with feasibility, acceptability, and efficacy in a population-based study in China. Encouragingly, our findings showed that smoking cessation supported by a mobile-phone-based text messaging intervention (Happy Quit), with either high- or low-frequency messages, for quitting smoking increased the quit rate at 24 weeks (biochemically verified continuous smoking abstinence at 24 weeks: 44/674 participants in the HFM group [6.5%], 17/284 participants in the LFM group [6.0%], and 8/411 participants [1.9%] in the control group). These findings suggest that the mobile-phone-based text messaging intervention (Happy Quit), with either high- or low-frequency messaging, was effective, and should be considered for large-scale use in China for intervention towards quitting smoking.

We identified no statistical difference in quit rate with the HFM intervention compared with the LFM intervention. Participants in the HFM group displayed higher self-reported continuous abstinence during the 12-week intervention period, but this quit rate decreased and was similar to the quit rate of those in the LFM group at the 24-week assessment point (Tables 2 and 3). For those who continued as smokers, participants in the HFM group, on average, reported 1 to 3 fewer cigarettes smoked per day than those in the LFM group over the course of the 24-week trial.

The 7-day point quit rates from week 1 to week 24 that we observed are roughly similar to those reported in other studies [16,23,24]; in our study they ranged from about 10% to more than 26% with intervention, and from less than 4% to almost 12% with no intervention. As noted earlier, however, smoking remains more highly normalized in China than in many other countries, and there are fewer generalized pressures to quit. It is interesting to note that the 7-day point prevalence cessation rate increased up to around 16 weeks and then reduced slightly in this trial. Other trials have reported similar patterns. For example, the Text2Quit program demonstrated that not smoking in the past 7 days increased in the intervention group from 30.5% at 1 month to 33.2% at 3 months and then slightly decreased to 31.7% at 6 months [15]. However, further studies are needed to explore the reasons for this pattern of a decreasing quit rate curve after 16 weeks: usually, with an imposed quit date, we would expect a continually increasing quit rate curve.

Although the intervention resulted in a 3-fold increase in biochemically verified continuous smoking abstinence at 24 weeks compared with control participants, the quit rate was relatively low. This low quit rate is not unique to our trial; a computer-tailored smoking cessation program in Switzerland evaluated about 2 decades ago, for example, demonstrated abstinence of 5.8% in the intervention group and 2.2% in the control group (*p <* 0.001) [25]. However, quit rates at the present time, on average, are relatively higher in European countries [13], where the denormalization of smoking has occurred in recent decades. Furthermore, the widespread practice of smoking in social networks may inhibit cessation among Chinese smokers, as not only do peers generally reinforce behaviors within networks but Chinese smokers embedded in networks of other smokers are more likely to have self-exempting beliefs that the negative effects of smoking will not befall them [26]. Thus, because Chinese smokers inhabit a social environment that is more supportive of smoking and less supportive of cessation, it is perhaps unsurprising that they may be more resistant to individual-level intervention than smokers in other countries. Yet, such intervention still makes a measurable difference relative to not receiving such assistance. A similar smoking cessation program conducted in Turkey (also a middle-income country) showed that CO-verified sustained abstinence at 3 months was 11% in the intervention group and 5% in the control group with intention-to-treat analyses [27]. Although this trial was underpowered, the available evidence suggested that its effects were similar to those of trials in other countries. A systematic review of RCTs with a pooled analysis showed no evidence of between-study heterogeneity (*I*^2^ = 0%). This review suggested that mobile-phone-based smoking cessation interventions significantly increased biochemically verified 7-day point prevalence of smoking cessation, with no evidence of increased adverse events [28].

Unlike some other countries like the United Kingdom and the United States where smoking cessation programs are well established and smoking cessation medications are widely available, the limited smoking cessation services and social support in China may partly explain the low quit rate we observed. In a study carried out in a Guangzhou smoking cessation clinic, for example, even though some participants from the clinic asked for nicotine replacement therapy or other medications for quitting, the clinic did not provide medications, as they were not available at that time. The point prevalence quit rate for that study at 6 months ranged from 18% to 24% during the follow-up period, which is similar to the findings in our current trial [29]. Furthermore, the advice from primary care providers on quitting smoking currently may not have a strong impact on Chinese smokers’ quitting or future intention to quit [30], but visiting a healthcare provider still has the potential to enhance attempts to quit smoking and promote abstinence [31]. Substantial work remains to be accomplished to assess the smoking cessation services provided by health professionals and quit rates in China.

The strengths of this trial include its large sample size from China and a particularly rigorous measure of abstinence with both self-reported response and biochemical verification, which is often considered the “gold standard” in validation studies. Furthermore, as far as we are aware, our trial represents the first RCT of a text messaging intervention for smoking cessation in China with long-term follow-up and biochemical confirmation for self-reported quitters. In addition, demographic data and smoking characteristics were evenly matched across groups.

This trial has some limitations, however. One limitation is that we could not prevent participants from using other smoking cessation services, although very few participants reported using them. Given the shortage of smoking cessation services in China, this may not be unusual. In China, male physicians tend to have a high smoking prevalence, and very few of them are former smokers. Also, standard smoking cessation tools and treatments are rarely provided by healthcare service providers [9]. Because the availability of smoking cessation training programs in China is extremely limited, most cessation attempts end in relapse [5]. Additionally, cotinine tests can only detect smoking status for a few days; thus, we sent several samples to self-reported quitters and required them to test at least twice between reporting days, and confirmed that the participant took the test via their significant other. We only evaluated the efficacy of the text messaging intervention for smoking cessation; studies are needed that compare the Happy Quit program to other established smoking cessation treatments, including hospital smoking cessation units, which are currently in use but with few clients in China. Although the Happy Quit program showed similar effects for younger and older adults, it is possible that the text messaging intervention may have a smaller effect for some underserved populations, such as HIV-positive or drug-using smokers. Yet, a randomized trial from the US displayed no long-term treatment effect for HIV-positive smokers [32]. Upon completion of the intervention trial, we offered the Happy Quit program booklet to all participants, but we do not know whether smokers in the control group who received the quit booklet after the trial also increased their quit rate afterwards. A previous study showed similar quit rates for participants with either the text messaging intervention or the quit booklet only [33]. By the end of our trial, the intervention groups had lower follow-up rates than the control group (83.2% of the HFM group, 74.6% of the LFM group, and 87.1% of the control group completed the study). Another limitation is the small proportion of quitters in this study. The relatively low quit rate might be a result of a shortage of other smoking cessation services in China; the challenging social context, in which behaviors of family members, friends, colleagues, and even health professionals may discourage smokers from quitting [34]; and the cultural norm of giving and sharing cigarettes in China [35]. Additionally, the participant recruitment for this trial did not meet the anticipated target sample size as specified in the protocol [18]. Nonetheless, based on our primary outcome (6.5% biochemically verified continuous abstinence at 24 weeks in the HFM group and 1.9% in the control group) and a 5% 2-sided type I error rate, a power of 80% requires a sample size of 610 and a power of 90% requires a sample size of 1,010. Thus, the sample size in the current trial should be considered reasonable. However, large-scale high-quality trials of the effects of optimized text message interventions on smoking cessation in different subgroups are warranted.

### Conclusions

Our findings indicate that the mobile-phone-based text messaging intervention Happy Quit, with messaging at either high frequency (on a daily basis) or low frequency (on a weekly basis), was successful in the present study, and can be considered for use in other settings in China. Because Happy Quit has far greater reach and higher feasibility among smokers than in-person treatments, and is not expected to have risks of adverse effects, it has the potential to improve population health and should be considered for inclusion in smoking cessation programs, to be made widely available for people who are seeking services for smoking cessation in China.

## Supporting information

S1 CONSORT Checklist (DOCX)

S1 Data Individual-level data. (XLSX)

S1 Fig Abstinence rates across the 3 groups at specified time points. *p < 0.05, **p < 0.01, ***p < 0.001. Self-reported abstinence was based on 7-day point prevalence (intention-to-treat).
(TIF)

S2 Fig Continuous abstinence rates across the 3 groups at 4 weeks, 12 weeks, and 24 weeks. *p < 0.05, **p < 0.01. Self-reported continuous abstinence at 4 weeks and 12 weeks and biochemically verified smoking cessation at 24 weeks.
(TIF)

S3 Fig Kaplan–Meier analysis of time to relapse (at 1, 4, 8, 12, 16, 20, and 24 weeks). Participants in the intervention groups had higher probability of continuous abstinence than those in the control group, especially during the follow-up period. The definition of relapse is smoking at least 5 cigarettes after the quit date (Russell Standard definition of relapse).
(TIF)

S1 Study Protocol (PDF)

S1 Table The geographical distribution of participants in China. (DOCX)

S2 Table Program acceptability. (DOCX)

S3 Table Baseline characteristics of study groups. (DOCX)

S4 Table Continuous smoking abstinence and 7-day point prevalence (intention-to-treat): Combined intervention group versus control group. (DOCX)

S1 Text Questions for assessing program acceptability. (DOCX)

## Abbreviations

CBT: cognitive behavioral therapy
FTND: Fagerstrom Test for Nicotine Dependence
HFM: high-frequency messaging
LFM: low-frequency messaging
OR: odds ratio
RCT: randomized controlled trial
SMS: short message service
