# Supplementary material for: Effectiveness of a text-messaging-based smoking cessation intervention (“Happy Quit”) for smoking cessation in China: A randomized controlled trial
Source: PLoS Med. 2018 Dec 18;15(12):e1002713. doi: 10.1371/journal.pmed.1002713 (PMC6298640; doi:10.1371/journal.pmed.1002713)
Supplement: S1 CONSORT Checklist — (DOCX) [file pmed.1002713.s001.docx]

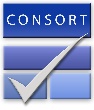
CONSORT 2010 checklist of information to include when reporting a randomised trial*

| Section/Topic | Item No | Checklist item | Reported on page No |
| --- | --- | --- | --- |
| Title and abstract | | | |
|  | 1a | Identification as a randomised trial in the title | See Title |
|  | 1b | Structured summary of trial design, methods, results, and conclusions (for specific guidance see CONSORT for abstracts) | See Author Summary |
| Introduction | | | |
| Background and objectives | 2a | Scientific background and explanation of rationale | See Introduction paragraphs 6 |
|  | 2b | Specific objectives or hypotheses | See Introduction paragraph 6 |
| Methods | | | |
| Trial design | 3a | Description of trial design (such as parallel, factorial) including allocation ratio | Methods paragraphs 3, 6 |
|  | 3b | Important changes to methods after trial commencement (such as eligibility criteria), with reasons | Methods paragraphs 6 |
| Participants | 4a | Eligibility criteria for participants | Methods paragraph 3 |
|  | 4b | Settings and locations where the data were collected | Methods paragraph 3 |
| Interventions | 5 | The interventions for each group with sufficient details to allow replication, including how and when they were actually administered | Methods paragraphs 3, 10-12 |
| Outcomes | 6a | Completely defined pre-specified primary and secondary outcome measures, including how and when they were assessed | Methods paragraph 13 |
|  | 6b | Any changes to trial outcomes after the trial commenced, with reasons | N/A |
| Sample size | 7a | How sample size was determined | Methods paragraph 14 |
|  | 7b | When applicable, explanation of any interim analyses and stopping guidelines | N/A |
| Randomisation: |  |  |  |
| Sequence generation | 8a | Method used to generate the random allocation sequence | Methods paragraph 7 |
|  | 8b | Type of randomisation; details of any restriction (such as blocking and block size) | Methods paragraph 6, 7 |
| Allocation concealment mechanism | 9 | Mechanism used to implement the random allocation sequence (such as sequentially numbered containers), describing any steps taken to conceal the sequence until interventions were assigned | Methods paragraph 7 |
| Implementation | 10 | Who generated the random allocation sequence, who enrolled participants, and who assigned participants to interventions | Methods paragraphs 7 |
| Blinding | 11a | If done, who was blinded after assignment to interventions (for example, participants, care providers, those assessing outcomes) and how | Methods paragraph 7 |
|  | 11b | If relevant, description of the similarity of interventions | N/A |
| Statistical methods | 12a | Statistical methods used to compare groups for primary and secondary outcomes | Methods paragraph 15, 16 |
|  | 12b | Methods for additional analyses, such as subgroup analyses and adjusted analyses | Methods paragraphs 16 |
| Results | | | |
| Participant flow (a diagram is strongly recommended) | 13a | For each group, the numbers of participants who were randomly assigned, received intended treatment, and were analysed for the primary outcome | Results paragraph 1, Figure 1 |
|  | 13b | For each group, losses and exclusions after randomisation, together with reasons | Figure 1 |
| Recruitment | 14a | Dates defining the periods of recruitment and follow-up | Results paragraph 1 |
|  | 14b | Why the trial ended or was stopped | N/A |
| Baseline data | 15 | A table showing baseline demographic and clinical characteristics for each group | Table 1  paragraph 2 |
| Numbers analysed | 16 | For each group, number of participants (denominator) included in each analysis and whether the analysis was by original assigned groups | Results paragraph 1, Figure 1 |
| Outcomes and estimation | 17a | For each primary and secondary outcome, results for each group, and the estimated effect size and its precision (such as 95% confidence interval) | Results paragraphs 3, 4, 5, Table 2 |
|  | 17b | For binary outcomes, presentation of both absolute and relative effect sizes is recommended | N/A |
| Ancillary analyses | 18 | Results of any other analyses performed, including subgroup analyses and adjusted analyses, distinguishing pre-specified from exploratory | Results paragraph 6, Tables 3 |
| Harms | 19 | All important harms or unintended effects in each group (for specific guidance see CONSORT for harms) | N/A |
| Discussion | | | |
| Limitations | 20 | Trial limitations, addressing sources of potential bias, imprecision, and, if relevant, multiplicity of analyses | Discussion paragraphs 7 |
| Generalisability | 21 | Generalisability (external validity, applicability) of the trial findings | Discussion paragraphs 8 |
| Interpretation | 22 | Interpretation consistent with results, balancing benefits and harms, and considering other relevant evidence | Discussion paragraphs 3, 4 |
| Other information | | |  |
| Registration | 23 | Registration number and name of trial registry | See Trial Registration (ClinicalTrials.gov, Identifier:NCT02693626) |
| Protocol | 24 | Where the full trial protocol can be accessed, if available | See S1 Study protocol.  Liao Y et al. BMC Public Health. 2016 Aug 19;16(1):833. |
| Funding | 25 | Sources of funding and other support (such as supply of drugs), role of funders | Funded by China Medical Board (CMB) Open (Grant Number 15-226).) |

*We strongly recommend reading this statement in conjunction with the CONSORT 2010 Explanation and Elaboration for important clarifications on all the items. If relevant, we also recommend reading CONSORT extensions for cluster randomised trials, non-inferiority and equivalence trials, non-pharmacological treatments, herbal interventions, and pragmatic trials. Additional extensions are forthcoming: for those and for up to date references relevant to this checklist, see [www.consort-statement.org](http://www.consort-statement.org).
