# Supplementary figures and images for: Effectiveness of a text-messaging-based smoking cessation intervention (“Happy Quit”) for smoking cessation in China: A randomized controlled trial
Source: PLoS Med. 2018 Dec 18;15(12):e1002713. doi: 10.1371/journal.pmed.1002713 (PMC6298640; doi:10.1371/journal.pmed.1002713)

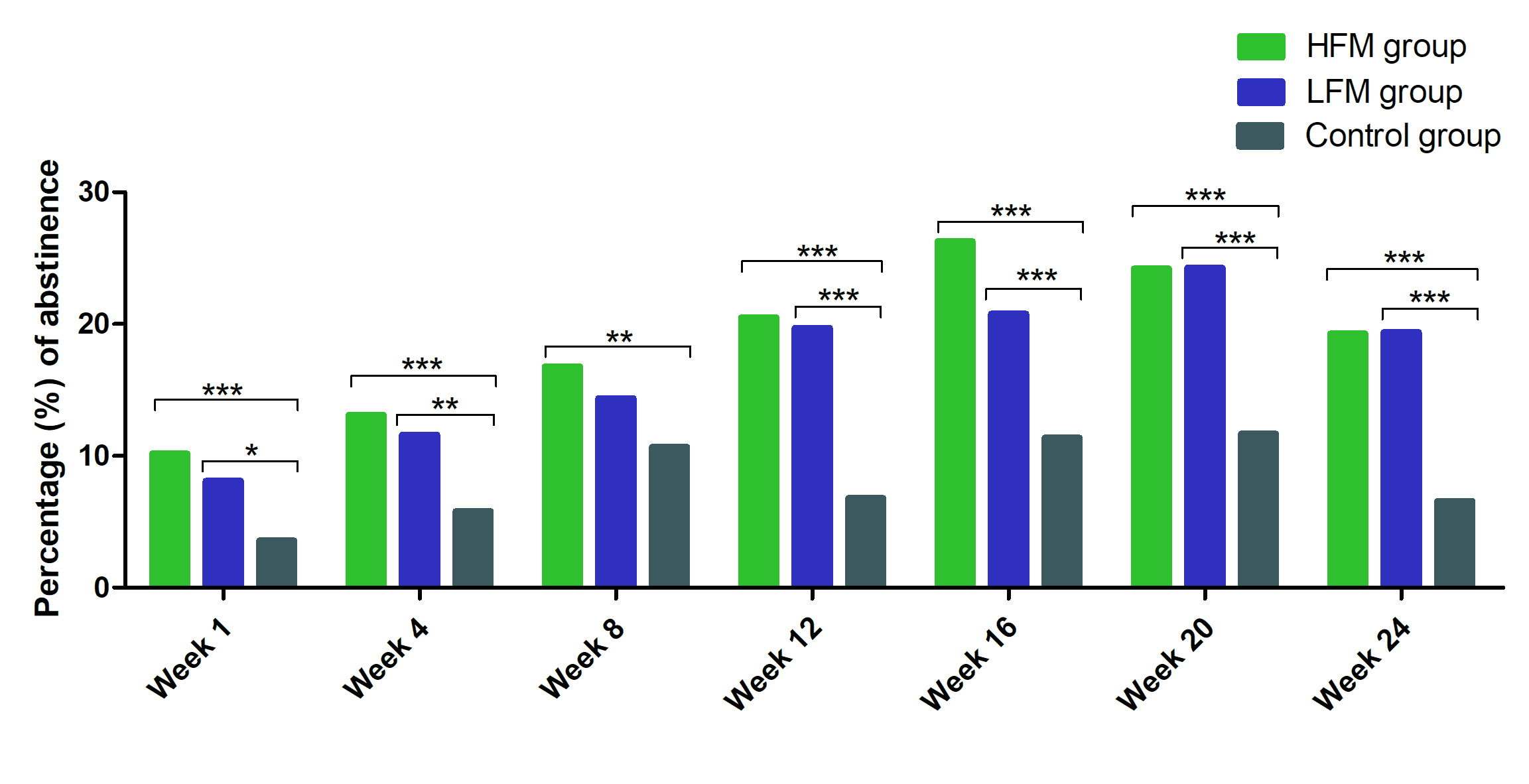

Supplement: S1 Fig — *p < 0.05, **p < 0.01, ***p < 0.001. Self-reported abstinence was based on 7-day point prevalence (intention-to-treat). (TIF) [file pmed.1002713.s003.tif]

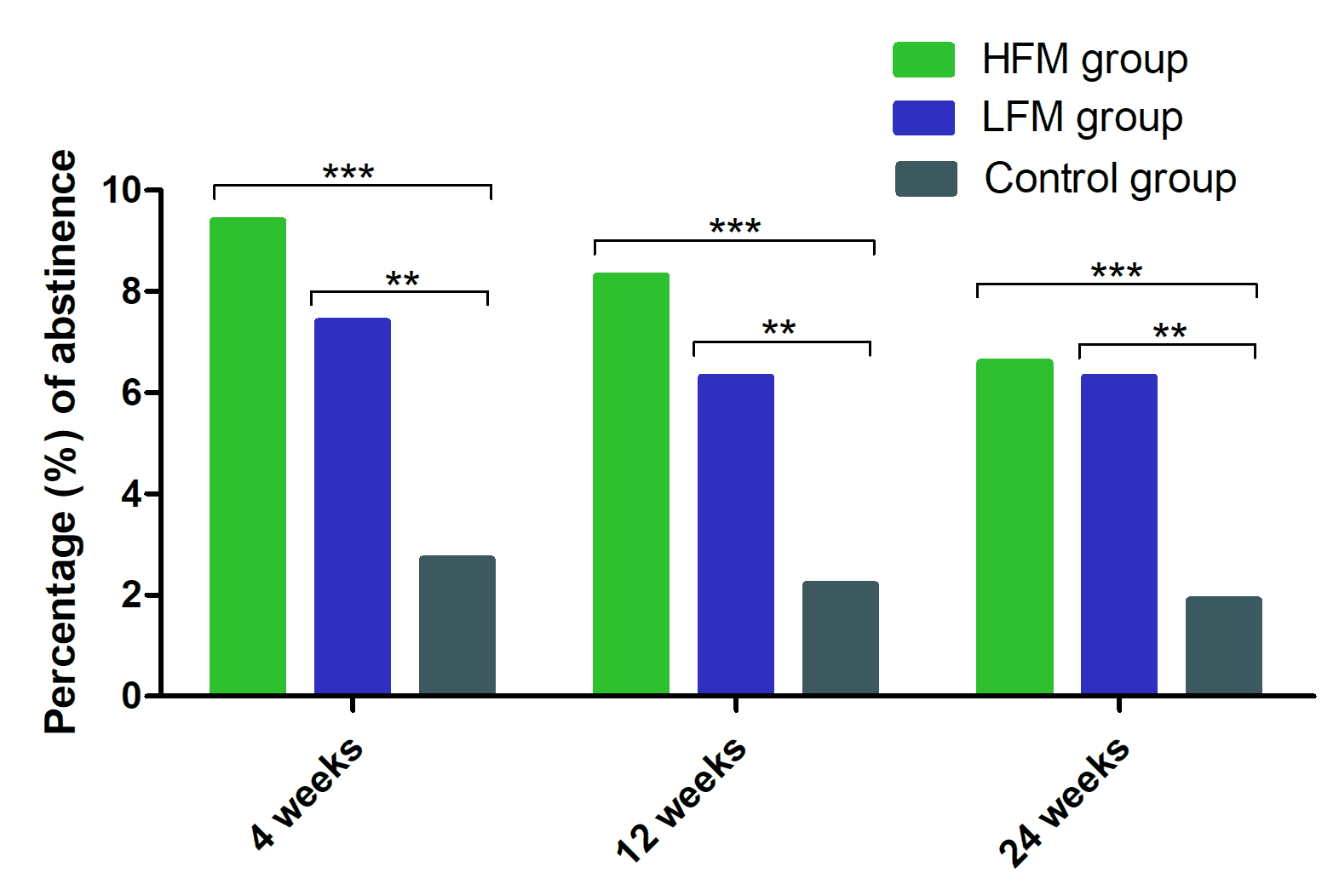

Supplement: S2 Fig — *p < 0.05, **p < 0.01. Self-reported continuous abstinence at 4 weeks and 12 weeks and biochemically verified smoking cessation at 24 weeks. (TIF) [file pmed.1002713.s004.tif]

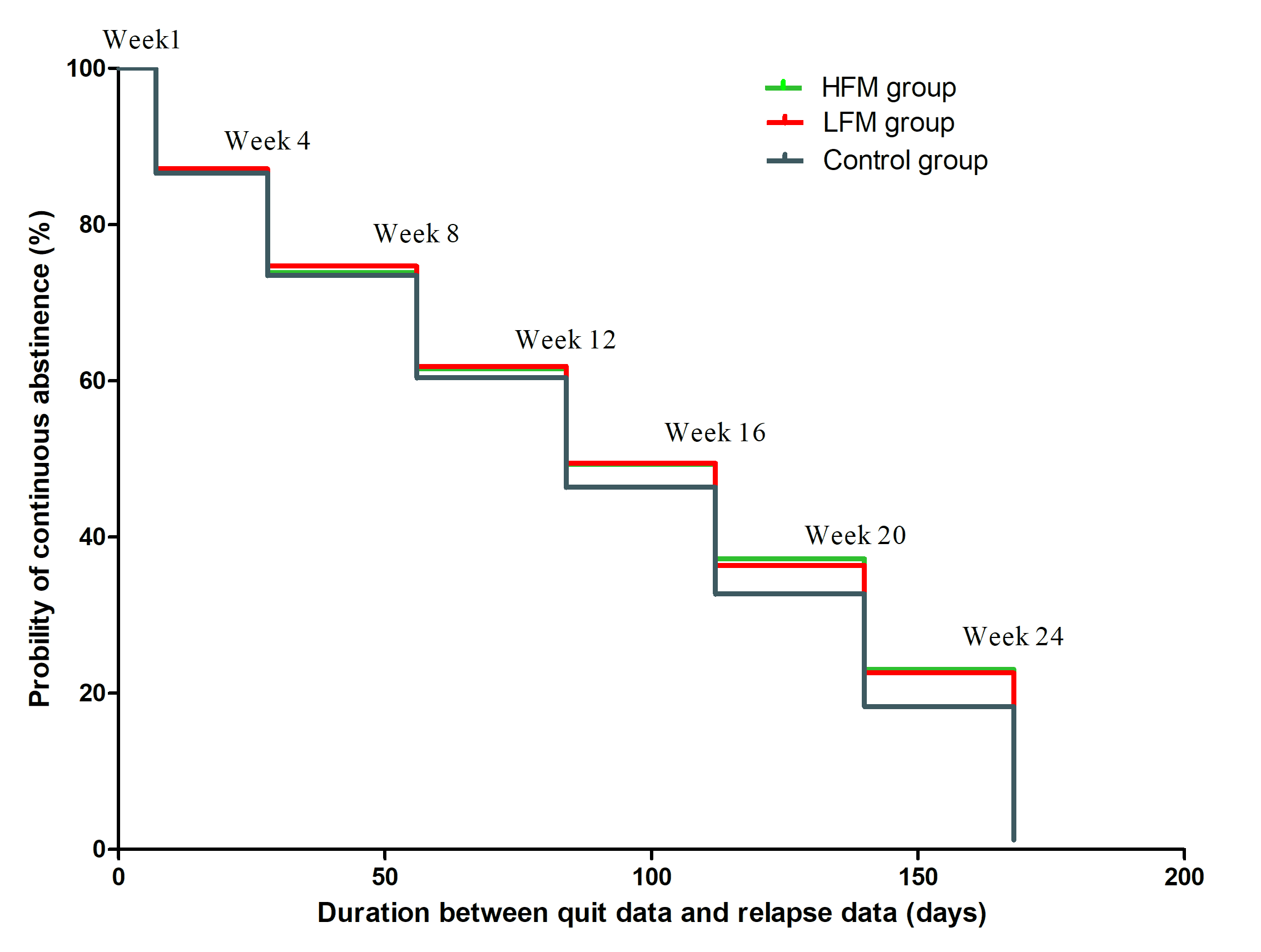

Supplement: S3 Fig — Participants in the intervention groups had higher probability of continuous abstinence than those in the control group, especially during the follow-up period. The definition of relapse is smoking at least 5 cigarettes after the quit date (Russell Standard definition of relapse). (TIF) [file pmed.1002713.s005.tif]
