# Supplementary material for: Effectiveness of a text-messaging-based smoking cessation intervention (“Happy Quit”) for smoking cessation in China: A randomized controlled trial
Source: PLoS Med. 2018 Dec 18;15(12):e1002713. doi: 10.1371/journal.pmed.1002713 (PMC6298640; doi:10.1371/journal.pmed.1002713)
Supplement: S1 Table — (DOCX) [file pmed.1002713.s007.docx]

**S1 Table. The geographical distribution of participants in China**

| Province/region | Number of subjects | % |
| --- | --- | --- |
| Hunan | 405 | 29.58 |
| Guangdong | 188 | 13.73 |
| Henan | 78 | 5.70 |
| Shandong | 58 | 4.24 |
| Jiangsu | 56 | 4.09 |
| Shanghai | 56 | 4.09 |
| Beijing | 53 | 3.87 |
| Yunnan | 47 | 3.43 |
| Hubei | 43 | 3.14 |
| Jiangxi | 40 | 2.92 |
| Zhejiang | 39 | 2.85 |
| Sichuan | 35 | 2.56 |
| Fujian | 29 | 2.12 |
| Chongqing | 26 | 1.90 |
| Shaanxi | 26 | 1.90 |
| Anhui | 25 | 1.83 |
| Guizhou | 25 | 1.83 |
| Guangxi | 22 | 1.61 |
| Hebei | 19 | 1.39 |
| Xinjiang | 18 | 1.31 |
| Liaoning | 13 | 0.95 |
| Hainan | 13 | 0.95 |
| Gansu | 13 | 0.95 |
| Shanxi | 10 | 0.73 |
| Inner Mongolia | 10 | 0.73 |
| Heilongjiang | 8 | 0.58 |
| Ningxia | 6 | 0.44 |
| Tianjin | 4 | 0.29 |
| Jilin | 2 | 0.15 |
| Qinghai | 2 | 0.15 |
