# Supplementary material for: Effectiveness of a text-messaging-based smoking cessation intervention (“Happy Quit”) for smoking cessation in China: A randomized controlled trial
Source: PLoS Med. 2018 Dec 18;15(12):e1002713. doi: 10.1371/journal.pmed.1002713 (PMC6298640; doi:10.1371/journal.pmed.1002713)
Supplement: S2 Table — (DOCX) [file pmed.1002713.s008.docx]

**S2 Table. Program acceptability**

|  |  | HFM Group (%) n=17 | LFM Group (%) n=17 |
| --- | --- | --- | --- |
| Appraisal of program Likelihood of recommending program to others | Very likely | 47.1 | 58.8 |
|  | Somewhat likely | 35.3 | 23.5 |
|  | Neutral | 11.8 | 11.8 |
|  | Unlikely | 5.9 | 5.9 |
|  | not at all likely | 0.0 | 0.0 |
| Overall rating of the program | Like very much | 35.3 | 41.2 |
|  | Like somewhat | 47.1 | 47.1 |
|  | Neutral | 5.9 | 11.8 |
|  | Dislike somewhat | 11.8 | 0.0 |
|  | Very dislike | 0.0 | 0.0 |
| The program made it easier to quit smoking | Strongly agree | 17.6 | 23.5 |
|  | Agree | 35.3 | 29.4 |
|  | Neutral | 29.4 | 23.5 |
|  | Disagree | 17.6 | 23.5 |
|  | Strongly disagree | 0.0 | 0.0 |
| The program disrupted my daily schedule | Strongly agree | 0.0 | 0.0 |
|  | Agree | 0.0 | 0.0 |
|  | Neutral | 11.8 | 29.4 |
|  | Disagree | 82.4 | 52.9 |
|  | Strongly disagree | 5.9 | 17.6 |
| I would not have been able to quit without the program | Strongly agree | 11.8 | 5.9 |
|  | Agree | 41.2 | 23.5 |
|  | Neutral | 29.4 | 41.2 |
|  | Disagree | 17.6 | 29.4 |
|  | Strongly disagree | 0.0 | 0.0 |
| I stopped reading the messages by the end of the program | Strongly agree | 5.9 | 5.9 |
|  | Agree | 41.2 | 5.9 |
|  | Neutral | 41.2 | 35.3 |
|  | Disagree | 11.8 | 47.1 |
|  | Strongly disagree | 0.0 | 5.9 |
| Messages were easy to understand | Strongly agree | 58.8 | 82.4 |
|  | Agree | 11.8 | 0.0 |
|  | Neutral | 23.5 | 11.8 |
|  | Disagree | 5.9 | 5.9 |
|  | Strongly disagree | 0.0 | 0.0 |
| The messages talked about what I was experiencing and feeling | Strongly agree | 17.6 | 17.6 |
|  | Agree | 64.7 | 52.9 |
|  | Neutral | 5.9 | 11.8 |
|  | Disagree | 11.8 | 17.6 |
|  | Strongly disagree | 0.0 | 0.0 |
| I received too many text messages (Appraisal of text messages) | Strongly agree | 5.9 | 0.0 |
|  | Agree | 17.6 | 0.0 |
|  | Neutral | 41.2 | 29.4 |
|  | Disagree | 29.4 | 58.8 |
|  | Strongly disagree | 5.9 | 11.8 |
| Frequency of reading text messages | Almost never | 0.0 | 0.0 |
|  | Sometimes | 94.1 | 11.8 |
|  | Always | 5.9 | 88.2 |

HFM: High-frequency messaging; LFM: Low-frequency messaging
