# Supplementary material for: Effectiveness of a text-messaging-based smoking cessation intervention (“Happy Quit”) for smoking cessation in China: A randomized controlled trial
Source: PLoS Med. 2018 Dec 18;15(12):e1002713. doi: 10.1371/journal.pmed.1002713 (PMC6298640; doi:10.1371/journal.pmed.1002713)
Supplement: S3 Table — (DOCX) [file pmed.1002713.s009.docx]

**S3 Table.** **Baseline characteristics of study groups**

|  | **Intervention group^*^**  **(n=958)** | **Control group**  **(n=411)** | |
| --- | --- | --- | --- |
| Sex (%) |  |  | |
| Male | 908 (94.8%) | 387 (94.2%) | |
| Female | 50 (5.2%) | 24 (5.8%) | |
| Age (years) | 37.8 (9.76) | 38.7 (9.83) | |
| Age (years) |  |  | |
| 18-34 | 382 (39.9%) | 156 (38.0%) | |
| 35+ | 576 (60.1%) | 255 (62.0%) | |
| Education (years) |  |  | |
| ≤12 | 240 (25.1%) | 109 (26.5%) | |
| >12 | 718 (74.9%) | 302 (73.5%) | |
| Average number of cigarettes smoked per day | 20.1 (9.30) | 20.0 (8.93) | |
| Average number of cigarettes smoked per day |  |  | |
| ≤10 | 167 (17.4%) | 77 (18.7%) | |
| 11-20 | 561 (58.6%) | 238 (57.9%) | |
| 21-30 | 156 (16.3%) | 64 (15.6%) | |
| ≥30 | 74 (7.7%) | 32 (7.8%) | |
| Previous quit attempts | 1.5 (4.29) | 1.6 (5.22) | |
| Previous quit attempts |  |  | |
| Never | 413 (43.1%) | 170 (41.4%) | |
| 1-5 times | 519 (54.2%) | 225 (54.7%) | |
| ≥ 6 times | 26 (2.7%) | 16 (3.9%) | |
| FTND score | 4.6 (2.16) | 4.6 (2.17) | |
| FTND score |  |  | |
| < 4 (minimally dependence) | 290 (30.3%) | 128 (31.1%) | |
| 4-6 (moderately dependence) | 469 (49.0%) | 203 (49.4%) | |
| > 6 (highly dependence) | 199 (20.8%) | 80 (19.5%) | |
| Data are n (%) or mean (SD)  FTND: Fagerstrom test for nicotine dependence  **^*^**Combined High-frequency messaging and low -frequency messaging groups | | |  |
