# Supplementary material for: Effectiveness of a text-messaging-based smoking cessation intervention (“Happy Quit”) for smoking cessation in China: A randomized controlled trial
Source: PLoS Med. 2018 Dec 18;15(12):e1002713. doi: 10.1371/journal.pmed.1002713 (PMC6298640; doi:10.1371/journal.pmed.1002713)
Supplement: S4 Table — (DOCX) [file pmed.1002713.s010.docx]

**S4 Table. Continuous smoking abstinence and 7-day point prevalence (intent-to-treat), intervention group versus control group**

|  | **Intervention group**^*^**(n=958)** | **Control group (n=411)** | **Odds Ratio (95% CI)** | **P value** |
| --- | --- | --- | --- | --- |
| **Primary outcome** |  |  |  |  |
| Biochemically verified continuous abstinence at 24 weeks | 61 (6.4%) | 8 (1.9%) | 3.43 (1.62-7.23) | 0.004 |
| **Secondary outcomes** |  |  |  |  |
| Self-reported continuous abstinence |  |  |  |  |
| 4-week | 84 (8.8%) | 11(2.7%) | 3.49 (1.84-6.62) | <0.001 |
| 12-week | 74 (7.7%) | 9 (2.2%) | 3.74 (1.85-7.54) | <0.001 |
| 24-week | 64 (6.7%) | 8 (1.9%) | 3.61 (1.71-7.59) | <0.001 |
| Self-reported 7-day point prevalence abstinence |  |  |  |  |
| 1-week | 92 (9.6%) | 15 (3.6%) | 2.80 (1.60-4.90) | <0.001 |
| 4-week | 121 (12.6%) | 24 (5.8%) | 2.33 (1.48-3.67) | <0.001 |
| 8-week | 154 (16.1%) | 44 (10.7%) | 1.59 (1.12-2.28) | 0.011 |
| 12-week | 194 (20.3%) | 28 (6.8%) | 3.47 (2.29-5.26) | <0.001 |
| 16-week | 236 (24.6%) | 47 (11.4%) | 2.53 (1.81-3.55) | <0.001 |
| 20-week | 232 (24.2%) | 48 (11.7%) | 2.42 (1.73-3.38) | <0.001 |
| 24-week | 185 (19.4%) | 27 (6.6%) | 3.40 (2.23-5.19) | <0.001 |
| Data are n (%); CI: confidence interval. P value: Difference Fisher’s exact P value  ^*^Combined High-frequency messaging and low -frequency messaging groups | | | | |
