# Supplementary material for: Effectiveness of a text-messaging-based smoking cessation intervention (“Happy Quit”) for smoking cessation in China: A randomized controlled trial
Source: PLoS Med. 2018 Dec 18;15(12):e1002713. doi: 10.1371/journal.pmed.1002713 (PMC6298640; doi:10.1371/journal.pmed.1002713)
Supplement: S1 Text — (DOCX) [file pmed.1002713.s011.docx]

**Questions for assessing program acceptability**

1. Appraisal of program Likelihood of recommending program to others

A. Very likely

B. Somewhat likely

C. Neutral

D. Unlikely

E. not at all likely

2. Overall rating of the program

A. Like very much

B. Like somewhat

C. Neutral

D. Dislike somewhat

E. Very dislike

3. The program made it easier to quit smoking

A. strongly agree

B. Agree

C. Neutral

D. Disagree

E. strongly disagree

4. The program disrupted my daily schedule

A. strongly agree

B. Agree

C. Neutral

D. Disagree

E. strongly disagree

5. I would not have been able to quit without the program

A. strongly agree

B. Agree

C. Neutral

D. Disagree

E. strongly disagree

6. Appraisal of text messages/Frequency of reading text messages

A. Almost never

B. Agree

C. Always

7. I received too many text messages

A. strongly agree

B. Agree

C. Neutral

D. Disagree

E. strongly disagree

8. I stopped reading the messages by the end of the program

A. strongly agree

B. Agree

C. Neutral

D. Disagree

E. strongly disagree

9. Messages were easy to understand

A. strongly agree

B. Agree

C. Neutral

D. Disagree

E. strongly disagree

10. The messages talked about what I was experiencing and feeling

A. strongly agree

B. Agree

C. Neutral

D. Disagree

E. strongly disagree
